# Motility enhancement of human spermatozoa using electrical stimulation in the nano-Ampere range with enzymatic biofuel cells

**DOI:** 10.1371/journal.pone.0228097

**Published:** 2020-02-20

**Authors:** Tai Eun Shin, Jin Woo Park, Won-Yong Jeon, Eun Ji Lee, Hyojeong Kwon, Boyoung Jeon, Hyo Eun Kang, Myung Joo Kim, Dae Keun Kim, Hyug-Han Kim, Jung Jae Ko, Jae Ho Lee

**Affiliations:** 1 CHA Fertility Center Seoul Station, Jung-gu, Seoul, Republic of Korea; 2 Department of Chemistry, College of Advanced Science, Dankook University, Anseo-dong, Cheonan, Republic of Korea; 3 School of Chemical Engineering, Sungkyunkwan University, Suwon, Republic of Korea; 4 Department of Urology, CHA Fertility Center, Seoul Station, CHA University, Seoul, Republic of Korea; 5 Department of Biomedical Science, College of Life Science, CHA University, Pochen, Gyounggi-do, Republic of Korea; University Hospital of Münster, GERMANY

## Abstract

Sperm motility is a crucial factor for normal fertilisation that is partly supported by mitochondrial activity. Enzymatic biofuel cells (EBFCs) generate electric currents by an electron grade from anodic to cathodic electrodes in a culture media. We demonstrate that electrical stimulation by EBFC at the nano-Ampere range enhances sperm motility that can potentially allow the development of a new therapeutic tool for male infertility, including poor motility. EBFC was set up with three different electrical currents (112 nA/cm^2^ and 250 nA/cm^2^) at two different times (1 h, 2 h). Each sample was evaluated for its motility by computer-assisted sperm analyses and sperm viability testing. In the expanded study, we used the optimal electrical current of the EBFC system to treat asthenozoospermia and sperm with 0% motility. Results showed that optimal electrical stimulation schemes with EBFCs enhanced sperm motility by 30–40% compared with controls. Activated spermatozoa led to tyrosine phosphorylation in the tail area of the sperm following the electrical stimulation in the nano-Ampere range. However, the electrically stimulated group did not exhibit increased acrosomal reaction rates compared with the control group. In cases related to asthenozoospermia, 40% of motility was recovered following the electrical stimulation at the nano-Ampere range. However, motility is not recovered in sperm with 0% motility. In conclusion, we found that sperm motility was enhanced by exposure to electrical currents in the nano-Ampere range induced by optimal EBFCs. Electrical stimulation enhanced the motility of the sperm though tyrosine phosphorylation in spermatozoa. Therefore, our results show that electrical currents in the nano-Ampere range can be potentially applied to male infertility therapy as enhancers of sperm motility in assisted reproductive technology.

## Introduction

Infertility is a common disorder with a prevalence of ~20% in all couples, while males comprise almost 40% of the infertility patients [1–3]. Male fertility is evaluated by semen analysis based on the guidelines defined by the World Health Organization (WHO) [4]. Adequate sperm motility is one of the most important factors for the traversal of sperm to the female genital tract and for the fertilisation of an oocyte. The required energy for the motility of the sperm is supported by the energy provided by the mitochondria that is mainly distributed at the mid-neck region. The energy generated by the mitochondria is used for the phosphorylation of flagellar proteins [5–7]. It initiates or maintains sperm motility that is essential for normal fertilisation. Currently, chemicals, such as pentoxifylline (a methylxanthine derivative) are used to enhance human sperm motility [8]. The pentoxifylline induces tyrosine phosphorylation of the intracellular sperm protein and leads to hyperactivation and increased motility [9]. Chemicals may enhance sperm motility artificially. The chemically treated sperms rapidly gain increased motility, but they lose this enhancement within 1 to 2 h [10]. Several reports have suggested that pentoxifylline is ineffective for enhancing motility in the clinical field [11, 12].

The motility of the sperm is associated with the adenosine triphosphate product (ATP) activity of the mitochondria and ATP supports the phosphorylation of sperm flagger protein like serine and tyrosine [6, 13]. The phosphorylation of tyrosine protein in the sperm tail onsets hyper-activation motility of the sperm for fertilization with oocytes [5]. However, energy loss partly occurs owing to the loss of mitochondrial function [14]. Therefore, optimal substrate support that provides an adequate number of electron donors is required to maintain and enhance sperm motility [15]. In theory, the introduction of a electrical current in the nano-Ampere range acts as an exogenous source of electrons, and serves as a biophysical source to enhance sperm motility [13]. There are a reported that electrical system dives utilized for sperm isolation for the in vitro fertilization[16]. The purpose of the electrophoretic system is to serve as an isolation system for normal sperm, such as for its decontamination from spermatozoa in the raw semen state [16]. However, the electrophoretic system was not focused on the enhancement of sperm motility in conjunction with the use of electrical stimulation.

Enzymatic biofuel cells (EBFC) have several advantages compared to non-organic fuel-cell systems. Specifically, bio-organic materials can be set up using simple methodologies, can easily control electrical currents in the nano- or microscale Ampere range, and are very economical for cellular electrical stimulation. The two components of the EBFC system include simple anodic and cathodic compartments. In the cathodic compartment, the bilirubin oxidase (BOD) consumes electrons via the reduction of oxygen (O_2_) to water (H_2_O). Hence, there is an electron-poor nano-environment in the vicinity of the cathode. In the anodic compartment, glucose oxidase (GOX) releases electrons via oxidation of glucose to gluconolactone by creating an electron-rich nano-environment in the vicinity of the anode [17, 18]. In a similar manner, the EBFC can create an electron gradient between the anodic and the cathodic enzyme compartments, thus resulting in an electrical current in the nano-Ampere range within the implanted region [18]. Therefore, the EBFC can be used at the cellular level as an electrical stimulation system without the need of an additional, dedicated device.

In this study, we investigated the possibility of utilisation of an EBFC system a) to enhance phosphorylation activity of tyrosine protein, and b) to increase the motility of human spermatozoa. We have also identified the optimal conditions to achieve these effects of EBFC as an external source of electrical stimulation for the ART.

## Materials and methods

### Preparation of enzymatic biofuel cells in culture dishes

**Fig 1:**
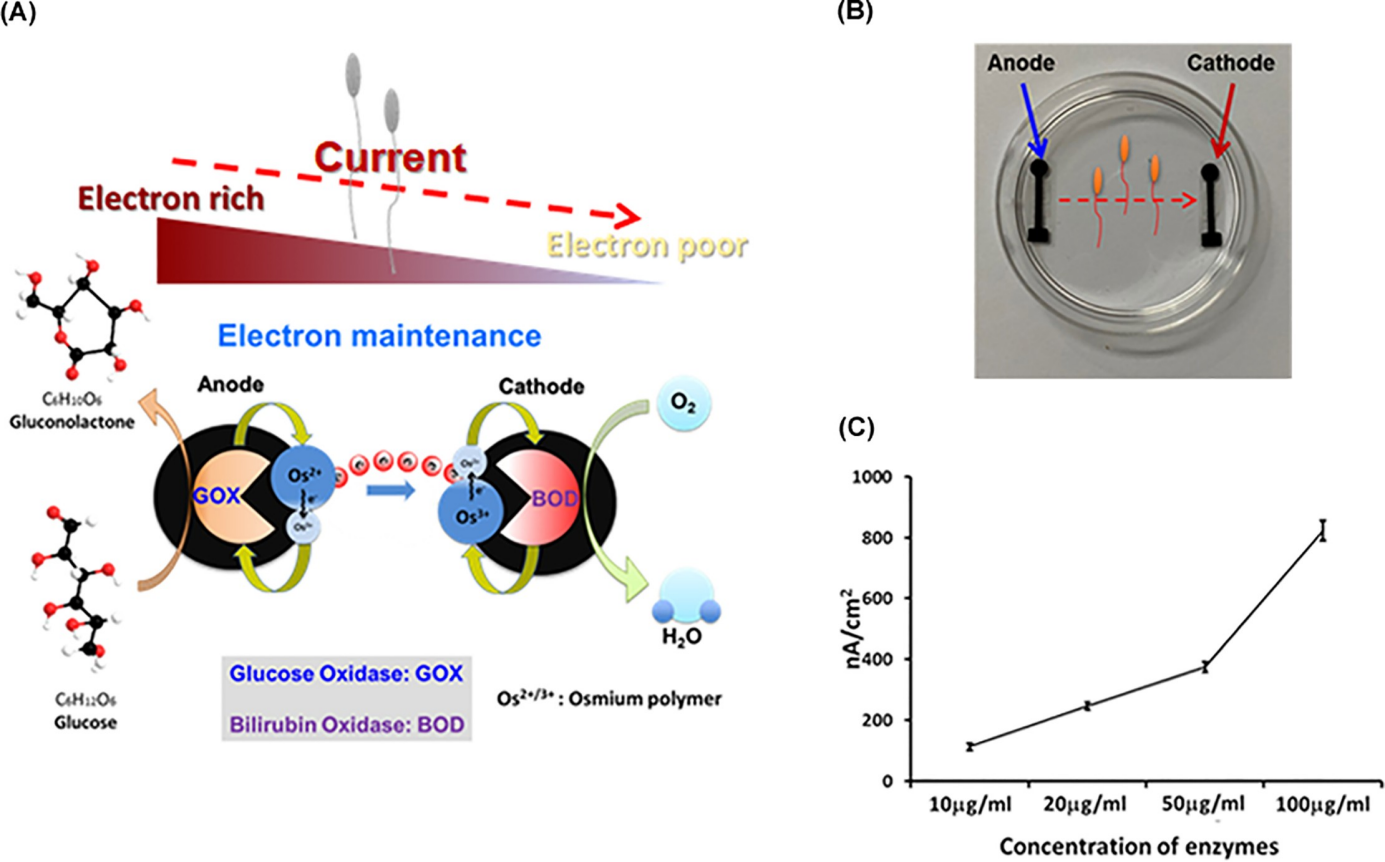
Principle and picture of enzymatic biofuel cell. (A) Enzymatic biofuel cells and electrical current analysis with culture media; (B) picture of enzymatic biofuel cell (EBFC) and electrical current on the 60 mm culture dish; (C) graph showing the current density of the EBFC at various enzyme concentrations (10, 20, 50, and 100 μg/mL) that generate different electrical currents in the media of the culture dish. All data are means ± standard errors of the means (SEM) of values acquired from three repeated analyses.

As shown in Fig 1A, EBFC has two different enzyme components which constitute a main core part. Glucose is oxidised by GOX in the anode electrode of EBFC and generates electrons. In the cathode electrode of EBFC, oxygen, hydrogen, and electron are transformed to water by BOD. Therefore, EBFC can generate the energy in this system. The mediator is an important component in the electron transfer from the enzyme to the electrode. Generally, Os, Ru, and Fe use redox electrode mediators as EBFCs or biosensors. Poly diglycidyl (ethylene glycol) has been used as a cross-linker to facilitate bonding with the enzyme, redox polymer, and electrode. We investigated the electric stimulation of cells, using EBFC. The GOX electrode was used in the culture media of spermatozoa as a rich-electron source, and the BOD electrode was used in the culture media of spermatozoa as a poor source of electrons. Finally, the full system was used in the culture media of spermatozoa for electron transport through the culture media of the dish (Fig 1B).

### Preparation of human spermatozoa

This study was approved by the Institutional Research and Ethical Committees of the CHA University, Korea (Institutional Review Board (IRB) numbers: 1044308–201610–BR–025–03). All the investigators underwent training and were certified for the study of human materials in biomedical research. Samples were collected and used for research investigations only after patient consent was obtained from heath male IVF patients. The study was undertaken after semen analyses. The collected semen was processed based on routine protocols of semen preparation, including swim-up methods. We used a total of 23 semen samples for the experiment: 19 sperm samples ranked as normal based on semen analysis criteria, 3 samples with severe asthenozoospermia, and 1 semen sample from the sperm with 0% motility. Each sample was divided in two groups: the control group with no electrical stimulation and other experimental group with electrical stimulation. Therefore, we observed sperm motility responses after the electrical stimulation to the sperm was terminated. We measured sperm motility 1 to 2 h after the termination of electrical stimulation of the incubation. Each experiment was evaluated three times for statistical analysis.

### Electrically stimulated normal and abnormal spermatozoa: A behavioural profile study

We performed motility analyses of spermatozoa with electrical stimulation via EBFC at 1 h, and 2 h. The count of spermatozoa was adjusted to range between 20–50 × 10^6^/mL concentration with Ham’s F10 nutrient mix (11550043, Gibco, Life technologies US), and was supplemented with 10% serum protein substitute (SPS: ART–3010, Quinn’s advantage serum protein substitute (SPS) kit, SAGE, US). The EBFC system was launched in a 60 mm culture dish (Fig 1A) which was filled with 3 mL Ham’s F10 with supplemental media (including 1000 mM glucose). The characteristics of sperm were analysed by computer-assisted sperm analysis (CASA), (CASA sperm class analyser ® CASA system version 6.2.0.15, microscopic SL, Barcelona, Spain). The CASA outcomes were based on the count, motility, and kinematic parameters (curvilinear velocity: VCL, linear velocity: VSL, average path velocity: VAP, and linearity: LIN). The samples were analysed three times with a 10× objective lens with positive phase-contrast microscopic imaging (ECLIPSE Ci/S, Nikon, Japan) with an area scan camera (acA780–75gc, Basler ace, Germany). And we performed the sperm viability assay using fast green and eosin staining though routine methods. We are using 0.9% sodium chloride, 1% eosin Y, 2% fast green mixture dye solution. Vital staining dye mixture with sample 1:1 ratios for 30sec, room temperature and smearing on the slide glass.

Total immotile sperm analyses were performed with microscopy at high-magnification settings. Sperm with 0% motility were transferred dropwise on the glass button dish whose cover was coated with mineral oil. We observed the dishes with high-magnification microscopy with an inverted microscope (ECLIPSE Ti2–E, Nikon, Japan) with a 6000× magnification. We analysed six different areas and quantified the head morphology of at least 100 spermatozoa per field using elliptic Fourier analysis. The morphological assessment of spermatozoa was based on the neck and tail shapes, the presence of mitochondria on the neck region, and on the tail’s morphology.

### Immunocytochemistry

We performed immunocytochemistry after electrical stimulation of human spermatozoa and washed and fixed them in cool methanol for 15 min at 4°C. Electrically stimulated spermatozoa were stained for intact acrosomal regions with pisum sativum agglutinin–fluorescein isothiocyanate dye (PSA–FITC conjugate: L0770, Sigma–Aldrich, US), and mitochondrial protein phosphorylation of sperm tail was detected using an anti-tyrosine phosphorylation antibody (anti-p-Tyr (pY99); SC-7020, Santa Cruz Biotech, USA). The primary antibody was an anti-pTyr(pY99) monoclonal mouse antibody in diluted phosphate buffer solution (PBS) with 0.01% bovine serum albumin (BSA), and was incubated 16hours at 4°C. The sample was then washed and treated for 1 h at room temperature with a secondary antibody, such as Cy5-labeled anti-mouse antibody with PSA–FITC (100 μg/mL). We finished the processing with nuclear count staining with 4’6’-diamidino-2-phenylindole (DAPI), and mounted the specimen with anti-fade solution (Vector shield® P100, USA) followed by imaging with a confocal microscope (ZISS M880, Germany). All images were processed and analysed for intensity of each red signal using the ZESS2016 software (ZEISS, Germany).

### Western blot

Electrically stimulated sperm was harvested immediately, centrifuged, and washed by PBS w/o Ca+ or Mg+. All samples were maintained at -80°C until the onset of the Western blot analyses. Each sperm sample protein was extracted with a protein lysis buffer (PRO-PREP^TM^ Cat. No 17081, iNtRon Biotechnology, Korea). The samples were boiled with 4×Laemmli sample buffer (Cat. No. 161–0747, Bio-Rad, CA, USA) for 5 min, and 20 μL of each boiled sample was loaded into the wells of an 8% sodium dodecyl sulfate polyacrylamide (SDS–PAGE) gel. We conducted the gel analyses at 80 V for 20 min, followed by 100 V at 30 min for the elution of the constituent proteins. The proteins were transblotted to a nitrocellulose (NC) membrane (Bio-Rad) at 350 mA for 2 h. The blotting NC membrane was incubated for 1 h with a blocking buffer (TBST with 5% BSA). The membrane was incubated with a primary antibody solution (anti-p-Tyr mouse antibody) overnight at 4°C. The blotted membrane was washed in TBS and incubated with horseradish peroxidase-conjugated anti-mouse immunoglobulin G (IgG) for 1 h at room temperature. Subsequently, the blotted membrane was washed with 0.5% tween-TBS and was incubated with a horseradish peroxidase-conjugated anti-mouse IgG secondary antibody (Bio-Rad). Immunoreactive bands were detected using an enhanced chemiluminescence (ECL) detection reagent (Clarity^TM^ Western blot substrate Cat.1705061, Bio-Rad, USA). The band image and intensity were analysed using the chemiluminescent imaging system (ATTO WSE–6100 LuminoGraph I, JP). The intensity of each sample band was analysed with the ImageSaver 6 version (ATTOS, Tokyo, JP) and was normalised with a beta-actin band intensity. The experiment was repeated three times with different samples, and was followed by the numerical analyses of each target band intensity ratio.

### Statistical analyses

All data are expressed as means ± standard error of the mean (SEM) of measurements in triplicate. Statistical analyses were carried out using one-way ANOVA test with a significance level set at *P* < 0.05. Significant differences are indicated by asterisks (*p* < 0.05) in each figures.

## Results

### Electrical property of EBFC and improved motility for human spermatozoa

**Fig 2:**
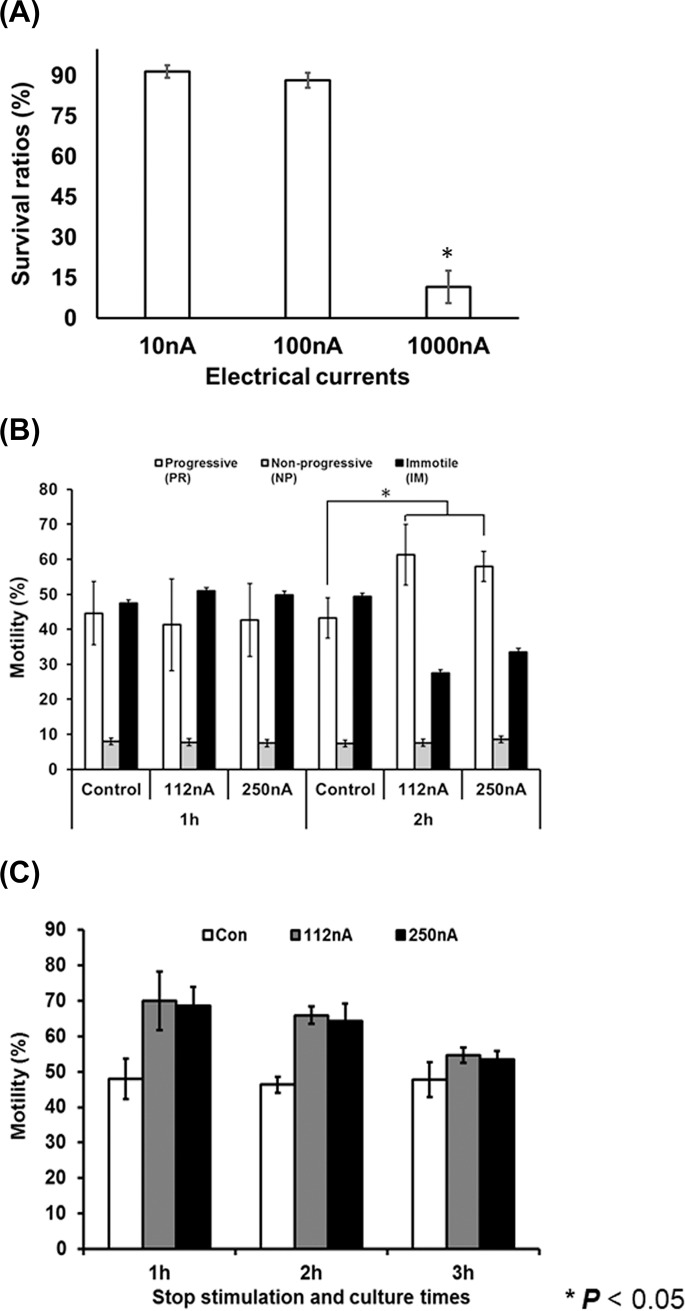
Optimal current range of electrical stimulation nano-Ampere range induced by EBFCs for spermatozoa survival and motility. (A) The bar graph presents the viability of the human spermatozoa with a specific electrical stimulation induced by the EBFC at currents in the range from 10 to 1000 nA/cm^2^. Currents in the range of 10 to 100 nA/cm^2^ do not induce sperm necrosis. However, currents of the order of 1000 nA/cm^2^ significantly decrease the sperm’s survival ratio compared to 100 nA/cm^2^ and in comparison, to the control group. (B) The bar graph presents the motility characterisation of human spermatozoa following electrical stimulation by EBFC at each studied time point. The open bar represents progressive (PR) motility, the gray bar represents the non-progressive (NP) motile sperm, and the black bar shows the immotile (IM) sperm with electrical stimulation. Electrical stimulation induced by the EBFC derives immotile, while none-progressive sperm derives into progressive sperm. (C) The bar graph represents the motility sperm ratios after the shutdown of electrical stimulation (1, 2 and 3 hours) ended. The open bar is the control, the gray bar corresponds to a current of 112 nA/cm^2^, and the black bar shows the electrical stimulation samples with a current of 250 nA/cm^2^. Stopping the electrical stimulation of the sperm shows decreased motility after 2 h in a manner similar to the response of the control sperm. All data are means ± SEM of values in triplicate. Significant differences are indicated by asterisks (* *p* < 0.05 against control).

The enzyme biofuel cell is composed of two major enzymes (BOX, GOD) and an osmium mixture which serves as the electrical conductor in the media. Enzyme biofuel cells generate various electrical current ratios depending on the enzyme concentration which ranges from 100 to 1000 nA/cm^2^ (Fig 1C). Bio-electrical current densities equal to 1000 nA/cm^2^ are obtained at high-enzyme concentrations. First, we evaluated sperm viability to determine the optimal electrical current (Fig 2A). The anode and cathode generated an electrical current density in the dish that was equal to 100 nA/cm^2^. As shown in Fig 2A, the electrical current (100 nA/cm^2^) had no toxicity effects. However, electrical currents in the range of 1000 nA were lethal within 1 h. These results show that an increased level of electrical current density > 500 nA/cm^2^ may cause electrical shock. To enhance the motility of the sperm, the optimal electrical current range of EBFC was lower than 500 nA/cm^2^. Fig 2 shows the variations of sperm (A) and motility (B) with the use of electrical stimulation by EBFC. Therefore, the optimal electrical stimulation of human spermatozoa occurred at 112 and 250 nA/cm^2^ in the 60 mm dish. The sperm motility was analysed by three patterns: progressive (PR), non-progressive (NP), and immotile (IM). Sperm motility PR ratios were not significantly enhanced in the electrically stimulated and control groups following 1 h incubation with EBFC. However, at 2 h, the electrical stimulation enhanced sperm motility from 20 to 30% compared with the control group (Fig 2B). Furthermore, ~30% decrease of immotile sperm was documented following electrical stimulation of the EBFC. Interestingly, the sperm motility at the cathode region was slightly higher than that at the anode. This suggests that rich electrical conditions induce increases in sperm motility compared with an electrically poor environment. We found that the EBFC system resulted in enhanced sperm motility in the electrical current range of 112nA and 250 nA/cm^2^. Therefore, Fig 2C presents the data collected after the shutdown of the electrical stimulation. Terminating the electrical stimulation of the sperm showed that the motility was maintained for 2 h. Subsequently, the motility ratio declined slowly in a similar manner to that observed in the cases of the control groups.

### Motility profile of human spermatozoa with optimal electrical stimulation

**Fig 3:**
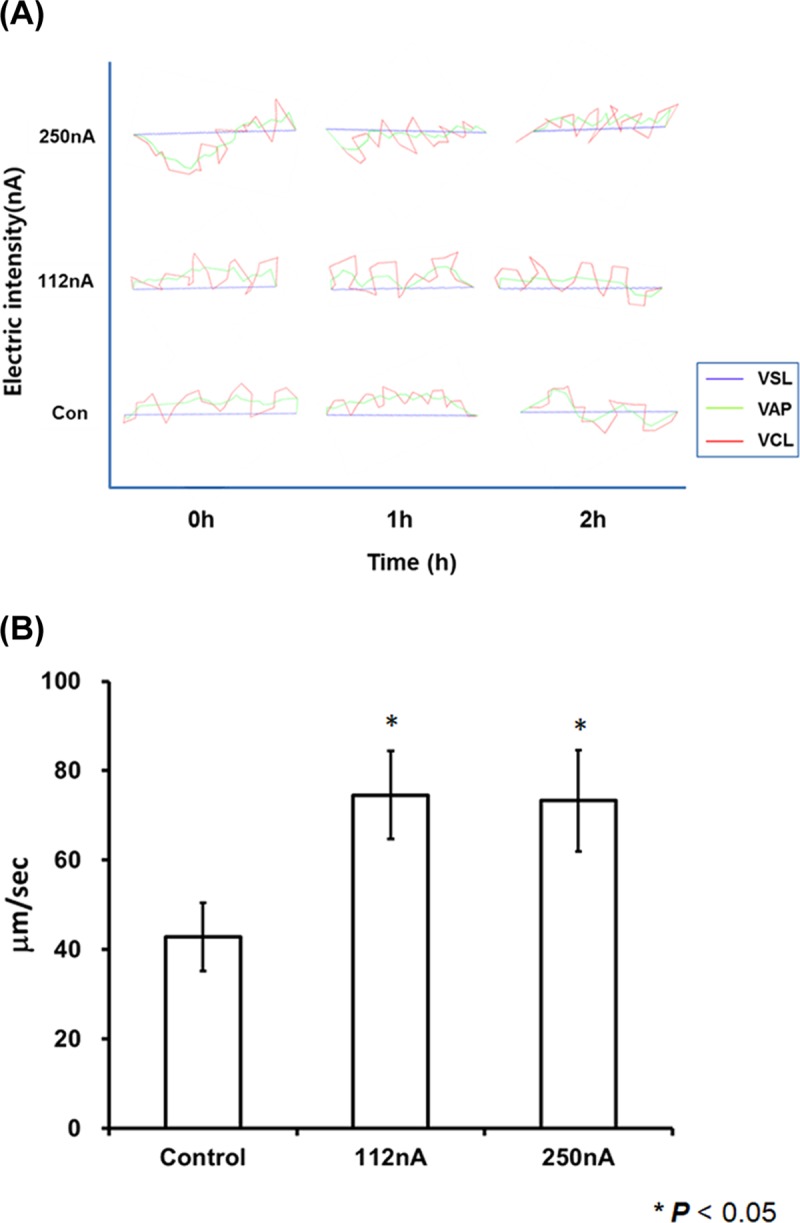
Computer-assisted sperm analysis of human spermatozoa at the initial timepoint, and at 1 h and 2 h following stimulation. (A) The line graph presents each sample denoting the VSL (purple), VAP (green), and VCL (red) values of electrically stimulated human sperm depending on the electrical current. (B) The moving distance per second of the control and the electrically stimulated human sperm: control, 112nA and 250nA/cm^2^ stimulation group. Both the 112 and 250 nA/cm^2^ electrical stimulation groups show significant increase in the straight movement and moving distance compared to the control group. All data are means ± SEM of values in triplicate. Significant differences are indicated by asterisks (*p < 0.05 against control).

Our CASA data show dependence on various factors related with the movement of electrically stimulated human spermatozoa ranging from immobility to none-progressive and progressive motilities (Fig 3A). Therefore, we evaluated the electrically stimulated sperm’s moving distance per second based on the movie clip of the CASA system. Both the 112nA and the 250nA electrical stimulated sperm exhibited movement that was two times faster (Fig 3B) and the straight tracking line pattern was also compared without an electrical stimulated group (S1, S2 and S3 Figs; S1, S2 and S3 Movies). This data suggests that the electrically stimulated sperm yields moving profile along straight lines depending on the exposure times and electrical current. As shown in Fig 3A and 3B, the sperm motility profiling was significantly increased, and exhibited a straight progressive moving pattern for a current density of 250 nA/cm^2^ following 2 h of electrical stimulation compared with the control group.

### Activation mechanism of electrically stimulated spermatozoa by EBFC

**Fig 4:**
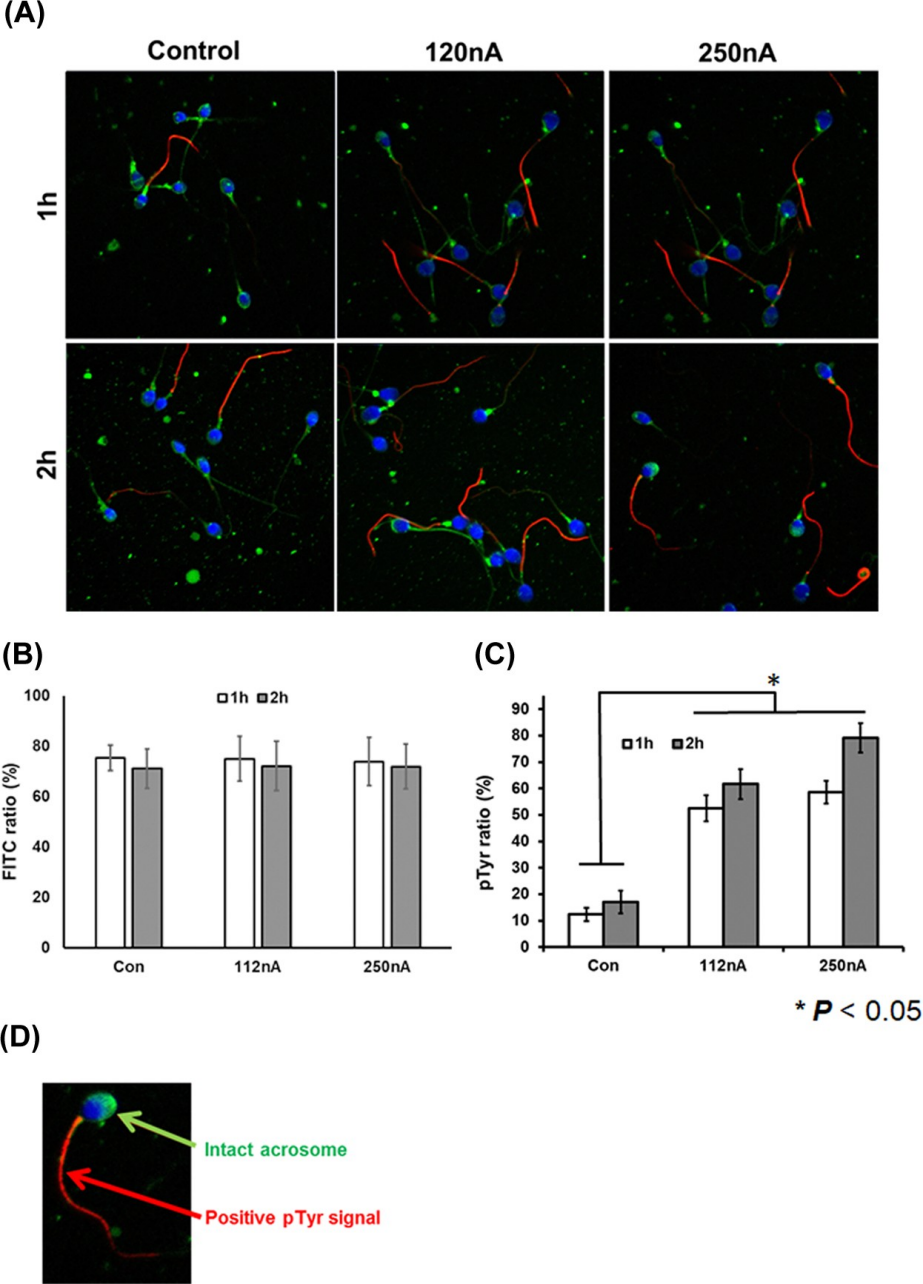
Immunocytochemistry of human spermatozoa following electrical stimulation induced by EBFC. (A) Confocal images of the electrical stimulation sperm stained with the pisum sativum agglutinin (PSA)–green fluorescent protein (FITC), p-Tyrosine protein antibody (red), and 4’6’-diamidino-2-phenylindole DAPI (blue) under 63 x magnifications. (B) The bar graph demonstrates the intact acrosomal ratios of the human spermatozoa in the electrical stimulation based on the GFP-positive signal. (C) The bar graph shows the positive p-Tyr staining signal ratios of the human spermatozoa in the electrical stimulation. The open bar denotes the samples following 1 h and 2 h (gray) of electrical stimulation. Electrical stimulation of sperm showed a strong positive signal of the tyrosine protein phosphorylation compared with the control group. In addition, the acrosomal status was not different between the control and electrical stimulation group. (D) A reprehensive confocal image indicating the intact acrosome and the p-Tyr positive sperm. All data are means ± SEM of values in triplicate. Significant differences are indicated by asterisks (* *p* < 0.05 against control).

**Fig 5:**
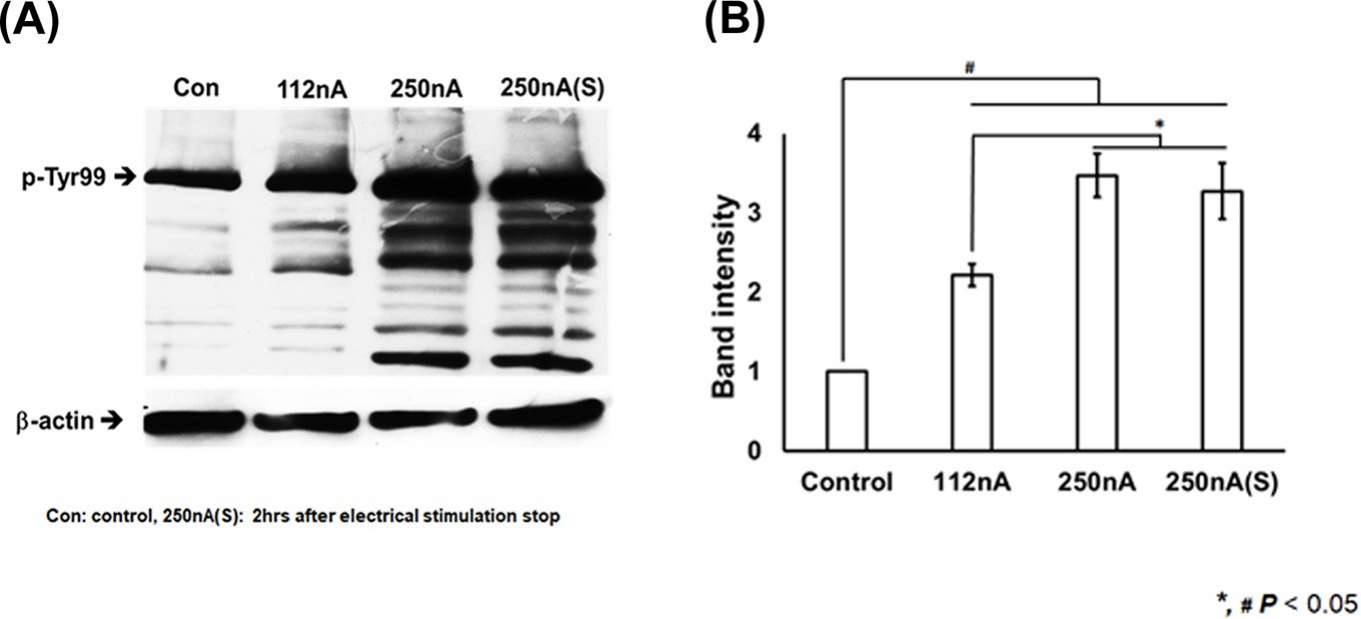
Quantification of tyrosine phosphorylation activity analysed by Western blot with the anti-p-Tyr antibody. (A) Immunoblot band image of phosphorylated tyrosine following the electrical stimulation of human spermatozoa with a normalisation band (con: control, 112nA, 250nA and 250nA(S): 2hrs after electrical stimulation stop, normalization by β-actin band). (B) The open bar graph shows the immunoblot band intensity obtained from the Western blot data. Electrically stimulated sperm showed a significant increase in the tyrosine phosphorylation ratios compared with the control group. All data are means ± SEM of measurements in triplicate. Significant differences are indicated by asterisks (*,# *p* < 0.05 compared with control).

To investigate whether increased phosphorylation activity of tyrosine protein in the sperm’s tail by electrical stimulation affects hyperactivation of sperm, we performed immunocytochemistry of the tyrosine phosphorylation ratios of the sperm (Fig 4) and quantified the outcomes with a Western blot with a p-Tyr antibody assay (Fig 5). These results show increased tyrosine phosphorylation activity in the electrically stimulated sperm (Fig 4A and 4C; supplement S6 and S7 Figs present full membrane image). However, acrosomal reaction was not induced within the electrical stimulation range (Fig 4B). Based on the immunocytochemical data, the amount of tyrosine phosphorylated spermatozoa was significantly increased in the electrically stimulated group by EBFC. The control group yielded a positive phosphorylation response in the range of 10–20%. However, electrically stimulated spermatozoa yielded a higher degree (70%) of positive phosphorylation signal following exposure to current densities in the range of 112–250 nA/cm^2^ (Fig 4C). Compared with the control group, tyrosine phosphorylation was increased two- to three-fold in electrically stimulated sperm compared with controls (Fig 5A). Tyrosine phosphorylation ratios was maintained and did not decrease until 2hrs even when electrical stimulation was stopped (Fig 5A and 5B).

### Motility recovery of asthenozoospermatozoa and the 0% motility sperm’s usage by EBFC

**Fig 6:**
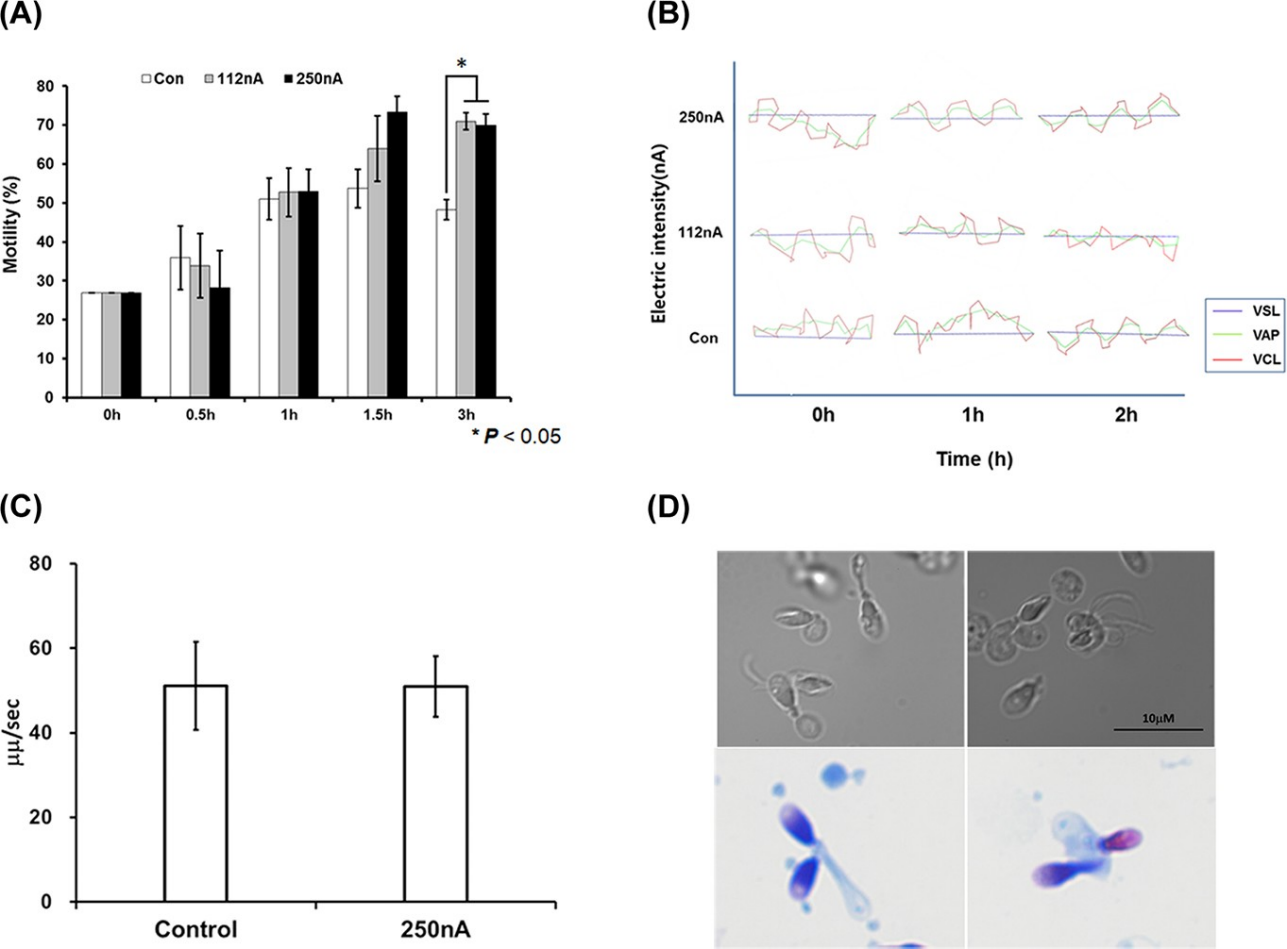
Recovery of motility of asthenozoospermatozoa and the immotile spermatozoa following electrical stimulation in the nano-Ampere range induced by EBFC. (A) The bar graph shows the electrically stimulated asthenozoospermatozoa motility ratios depending on the culture times which range from 0 h to 3 h. The open bar is the control group, the gray bar is the 112 nA/cm^2^ stimulation, and the black bar is the 250 nA/cm^2^ stimulation group. (B) The line graph presents the VSL (purple), VAP (green), and VCL (red) values of the electrically stimulated human asthenozoospermatozoa. (C) The moving distance per second of the control and electrical stimulated asthenozoospermatozoa sample. Control: control group, 250nA: 250 nA/cm^2^ stimulation group. The electrical stimulation of the EBFC to the asthenozoospermatozoa enhanced the sperm’s motility after 2 h and without a straight movement but no increased the distance moved by the sperm compared with the control groups. (D) Up side two images at high magnification at the different fields show immotile sperm morphology with neck and tail defects. And down side two images of strict morphology analysis clearly exhibits the neck and tail defect of immotile spermatozoa. Therefore, the defected immotile sperm evokes no responses following electrical stimulation. All data are means ± SEM of measurements in triplicate. Significant differences are indicated by asterisks (* *p* < 0.05 against control).

As shown in Fig 6A, the motility of asthenozoospermatozoa was increased following electrical stimulation with EBFC. The motility was enhanced in the control and electrically stimulated groups after washing with sperm media for 1 h. In particular, electrically stimulated sperm showed a significantly increased motility response compared to the control group after incubation for 3 h (Fig 6A). Additionally, the tracking line of the asthenozoospermatozoa utilized the movie clip of the CASA system exhibiting moving patterns between control and nano-ampere electrical stimulation groups (Figs 6B and S5). Fig 6B showed electrically stimulated asthenozoospermatozoa was similar to the straight mobility pattern compared with the control group (Fig 6B). Then, we measured the motile sperm’s moving distance per second through the CASA system. Also, electrical stimulation has not significantly enhanced the straight movement pattern compare to the control (Figs 6B; S4 and S5 Movies).

We then applied the electrical stimulation to the sperm with 0% motility to recover motility (Table 1). Electrical stimulation (250nA, 2hrs) with EBFC yields no rescue effects in the case of immotile sperm, even in the case of the sperm having an estimated percentage of 10% to survive. In Fig 6D, the immotile sperm shows an abnormally short tail and abnormal neck morphology, as evaluated with high-magnification microscopy (upper picture) and strict morphology analysis (down side picture). Therefore, immotile sperm does not respond to electrical stimulation, which is useful for rescue motility for sperm of neck and tail defect (Fig 6D).

**Table 1:** Profile of 0% motile spermatozoa and response of 250nA, 2hrs by electrical stimulation nano-Ampere range of EBFC.

	Count(X106/mL)	Motility(%)	Progressive Motility(%)	Survival ratios(%)
**Initial**	26.49±1.52	0	0	10±2.21
**2hrs**	26.49±1.52	0	0	9±3.21

## Discussion

In this study, we found that optimal electrical stimulation of the nano-Ampere range induced by EBFC enhanced sperm motility. Optimal electrical current promoted sperm motility that ranged from low-movement patterns to rapid movements, including hyperactivation and straight movement. Sperm motility requires ATP energises in mitochondria but also mechanical regulation of the fibrous sheath for the strong planar beat of flagellum. Our immunocytochemistry data show that the phosphorylation of tyrosine is increased in the electrically stimulated group. Therefore, the electrical stimulation of EBFC did not inducing acrosome reactions at the sperm’s head.

The regulation mechanism of sperm motility is still completely unidentified in regards to its factors and signal pathway in the mammalian. Especially, the mitochondria’s bioenergy production of the sperm is still debated on what system is involved for aerobic respiration. Normal activation processes of sperm motility requests optimal energy and activation of flagellar protein like dyneins in the tail by phosphorylation by kinase like the PKA. Major energy source of sperm motility is ATP from the product of mitochondria aerobic respiration. ATP produced by the mitochondria is converted to cyclic adenosine monophosphate (cAMP) that induces the activation of protein kinase A (PKA)[5]. Our hypothesis is that optimal nano-ampere electrical current drives the mitochondria to its aerobic respiration activity. Then, the high amount of the ATP product from sperm mitochondria promotes tyrosine phosphorylation ratios for sperm motility compared to the control group. Electrical stimulated sperm shows a high phosphorylation ratios of tyrosine protein. Sperm motility is enhanced by tyrosine phosphorylation in the regulation pathway. Tyrosine phosphorylation of sperm flagellar proteins has also been associated with the starting point and the end point as optimal sperm motility for hyperactivated sperm motility.

Based on the CASA movie clip, the electrical stimulated sperm shows more line movement pattern than the control group. Sperm has two different motility patterns depending on the female reproductive tract: a) sperm needs to be transported through the cervix area, and b) the hyperactivated motility is required for fertilization in the oviduct tubule region [6]. A straight movement pattern of the sperm is important for the penetration of zona pellucida for normal fertilization with oocytes. 112nA and 250nA nano-ampere electrical stimulation sperm shows a straight moving pattern and a 50% high speed ratio compared to the control group. Therefore, EBFC could be utilised for the enhancement of poor hyperactivated sperm for in vitro fertilization.

Asthenozoospermia exhibits low motility or immotile phenotypes, and prevents natural fertilisation [19]. To-this-date, the original cause of asthenozoospermia has not been elucidated. This has limited the clinical treatments, such as the intra-cytoplasmic sperm injection for poor sperm motility or the immotile sperm [20]. The motility of asthenozoospermatozoa correlated with mitochondrial bioenergy (ATP) generated via oxidative phosphorylation based on the utilisation of the electron transport system [21]. Ultimately, the potential difference of the membrane operates the ATP synthase complex and generates most of the energy used for sperm motility. In this process, the electron supply is an important requirement for ATP synthesis through several types of enzymes (Nicotinamide adenine dinucleotide and flavin adenine dinucleotide) related to electron transport at the membrane of the mitochondria [14]. Mitochondrial dysfunction reduces aerobic energy production and leads to changes at the tissue level depending on metabolic demands. Poor motility of the spermatozoa showed mtDNA mutation and deletion at the respiratory chain of complex I and II in the mitochondria membrane [22, 23]. Functional mitochondrial activity of mitochondria is related with poor motility of asthenozoospermia depend on the energy production activity [22]. Therefore, electrical stimulation of EBFC support to cellular mitochondrial membrane generates bioenergy of sperm. It is similar to that induced by a bioelectrical-mimic system operating with electrical current in the nano-Ampere range [24]. EBFC induces simple electrical currents in the nano-Ampere range depending on the enzyme concentration in vitro and in vivo [18, 25]. In the case of 0% motile sperm, the EBFC did not affect the recovery of the motility because they loss functional mitochondria bioenergy action. The electrical stimulation of the EBFC has a limited recovery percentage of sperm immotility that fails to support the amount of energy support required for motility. Therefore, 0% motile sperm a need to conduct additional studies to identify the role and recovery factor for the metabolic or the tail defect of the immotile sperm. Electrical stimulation may require the normal sperm with a functional mitochondrion and the tail for enhanced sperm motility.

## Conclusion

We found that the electrical stimulation of EBFC enhances human spermatozoa motility. Electrical stimulation in the nano-Ampere range with EBFC enhanced sperm motility via tyrosine phosphorylation without inducing acrosomal reactions. Electrical stimulated sperm induced progressive and straight sperm movement patterns. EBFC is a potential new strategy for the treatment of male infertility, and is considered to a potential therapeutic tool for poor motile sperm.

## Supporting information

S1 Fig Controls: No stimulated sperm movement. (TIF)

S2 Fig 112nA stimulated sperm movement. (TIF)

S3 Fig 225nA stimulated sperm movement. (TIF)

S4 Fig Control of asthenozoosperm. (TIF)

S5 Fig 250nA asthenozoosperm. (TIF)

S6 Fig Western blot full membrane image of pTyr99. (TIF)

S7 Fig Beta-actin full membrane image. (TIF)

S1 Movie Control: No stimulated sperm moving pattern. (MP4)

S2 Movie 112nA stimulated sperm moving pattern. (MP4)

S3 Movie 250nA stimulated sperm moving pattern. (MP4)

S4 Movie Control: No stimulated sperm moving pattern. (MP4)

S5 Movie 250nA stimulated asthenozoosperm moving pattern. (MP4)
