# Supplementary figures and images for: Motility enhancement of human spermatozoa using electrical stimulation in the nano-Ampere range with enzymatic biofuel cells
Source: PLoS One. 2020 Feb 20;15(2):e0228097. doi: 10.1371/journal.pone.0228097 (PMC7032714; doi:10.1371/journal.pone.0228097)

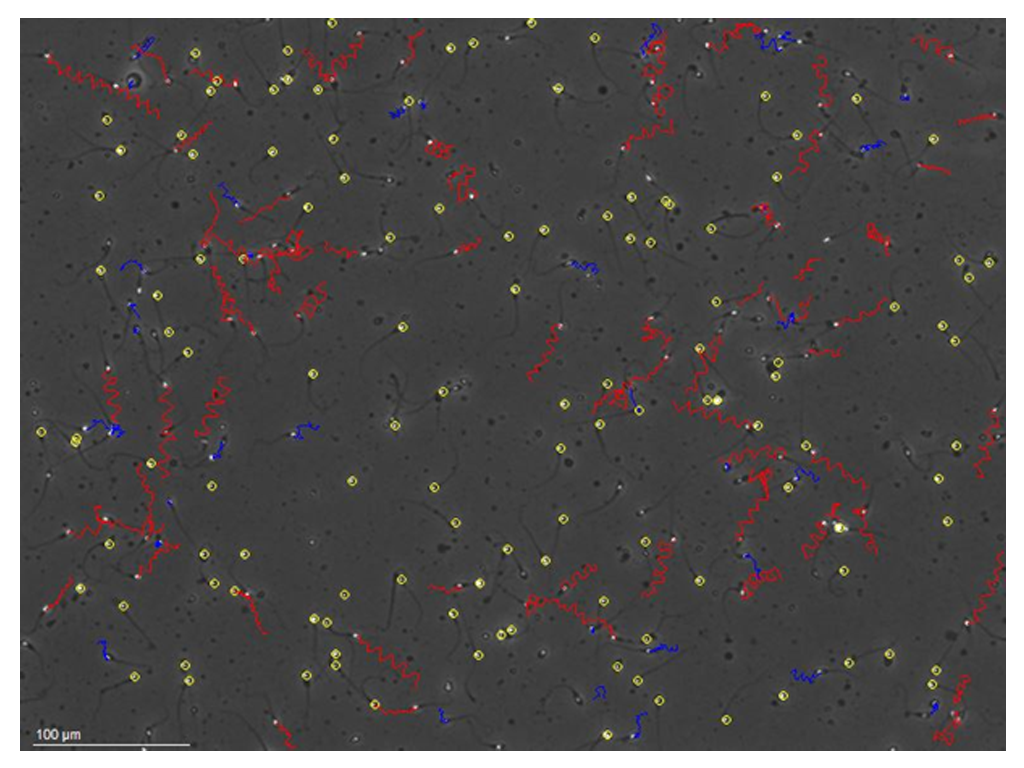

Supplement: S1 Fig — (TIF) [file pone.0228097.s001.tif]

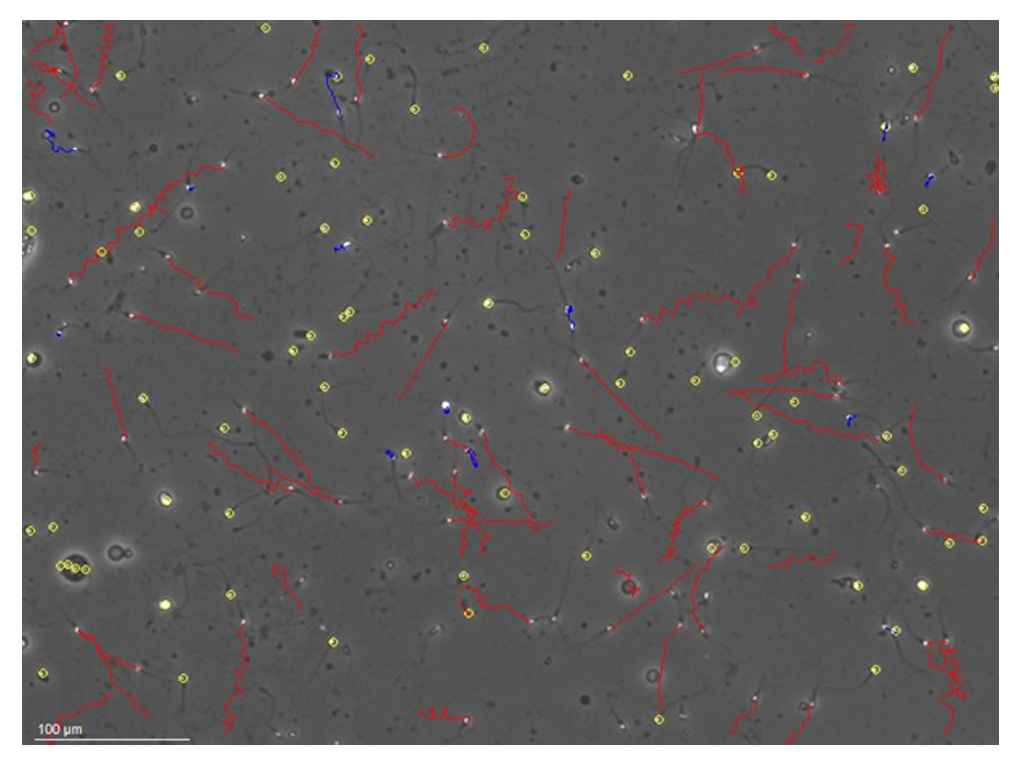

Supplement: S2 Fig — (TIF) [file pone.0228097.s002.tif]

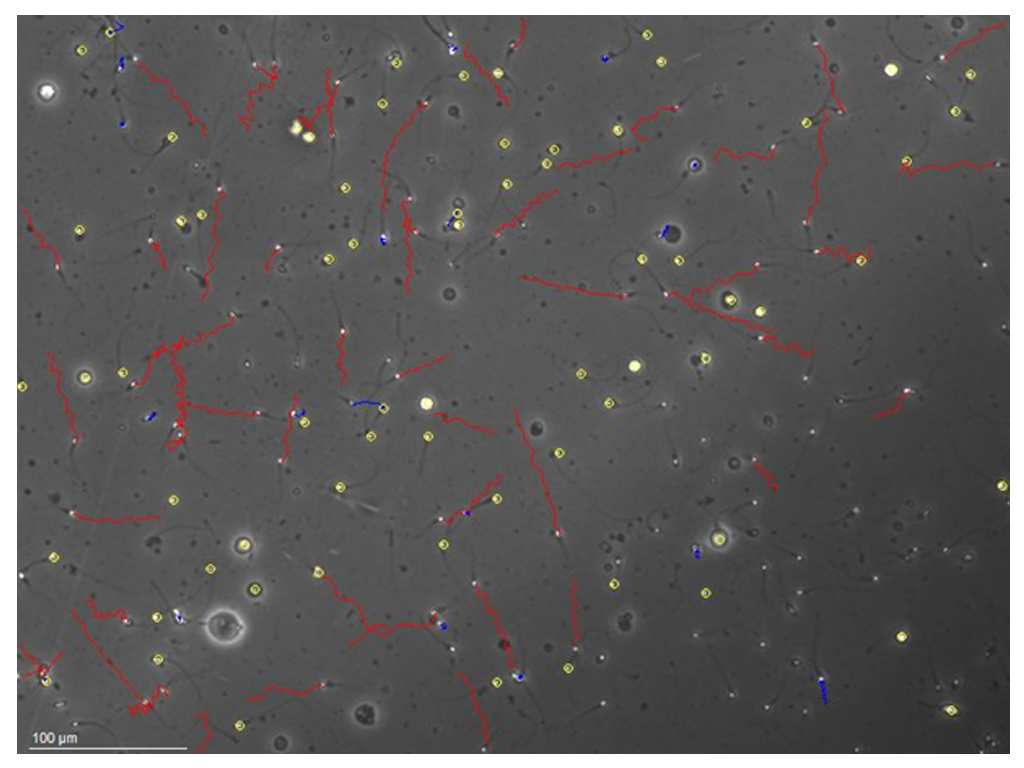

Supplement: S3 Fig — (TIF) [file pone.0228097.s003.tif]

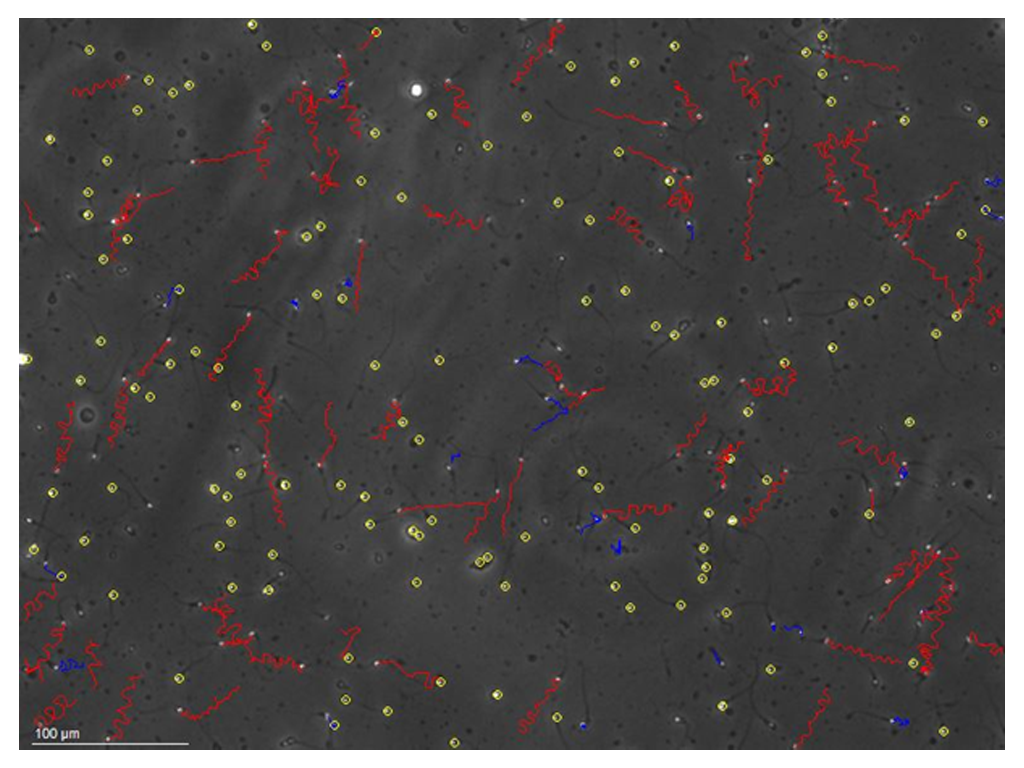

Supplement: S4 Fig — (TIF) [file pone.0228097.s004.tif]

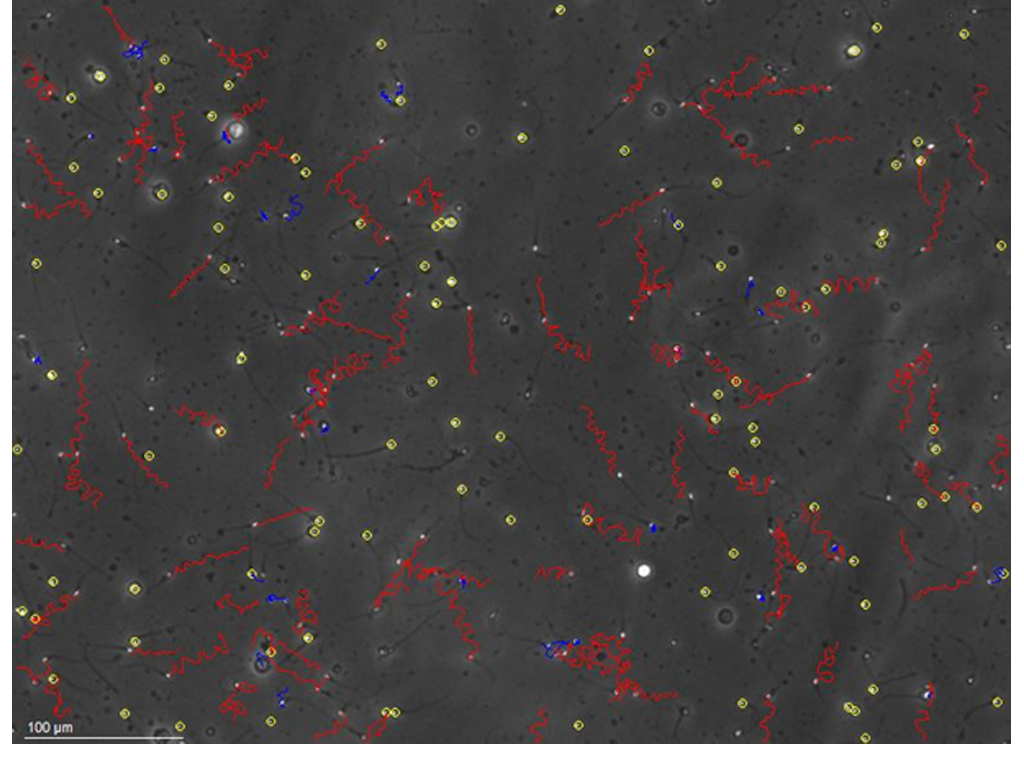

Supplement: S5 Fig — (TIF) [file pone.0228097.s005.tif]

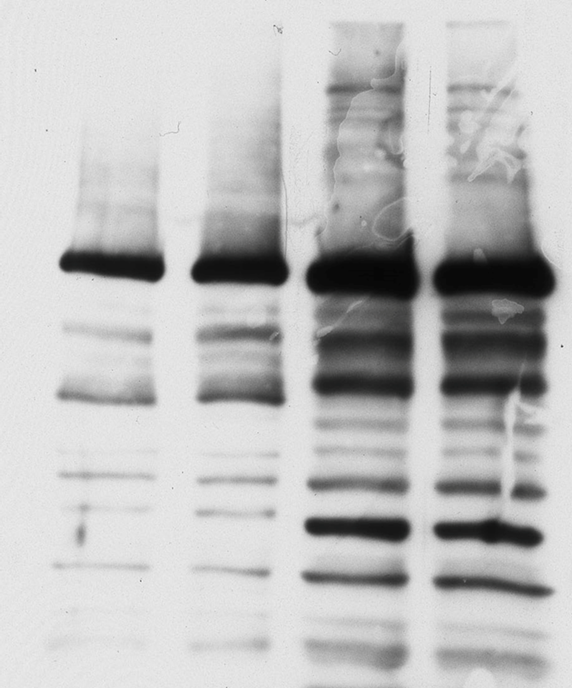

Supplement: S6 Fig — (TIF) [file pone.0228097.s006.tif]

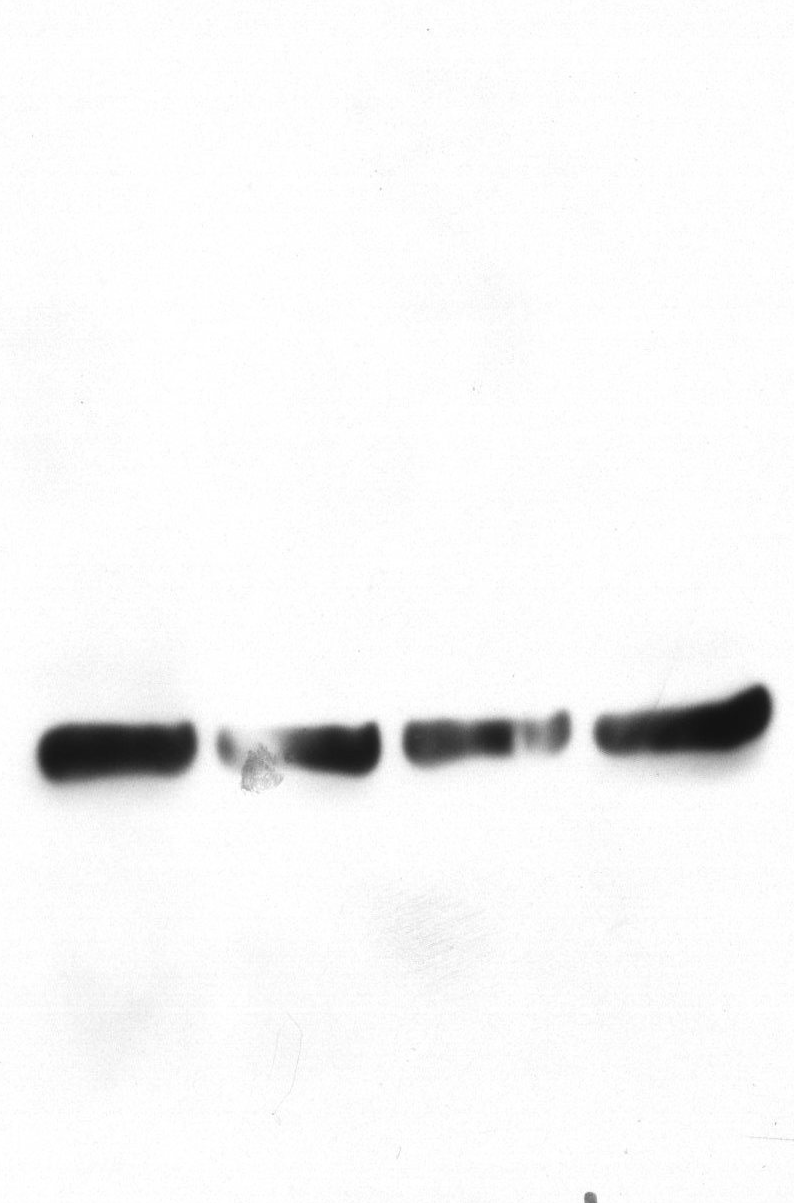

Supplement: S7 Fig — (TIF) [file pone.0228097.s007.tif]
